# Solid-State Solar Energy Conversion from WO_3_ Nano and Microstructures with Charge Transportation and Light-Scattering Characteristics

**DOI:** 10.3390/nano9121797

**Published:** 2019-12-17

**Authors:** Juyoung Moon, Woojun Shin, Jung Tae Park, Hongje Jang

**Affiliations:** 1 Department of Chemical Engineering, Konkuk University, 120 Neungdong-ro, Gwangjin-gu, Seoul 05029, Korea; 2 Department of Chemistry, Kwangwoon University, 20 Gwangwoon-ro, Nowon-gu, Seoul 01897, Korea

**Keywords:** solid-state electrolyte, tungsten oxide (WO_3_), hydrothermal, charge transportation, solar energy conversion, light scattering, dye-sensitized solar cell

## Abstract

Solar energy conversion devices composed of highly crystalline gel polymers with disk-WO_3_ nanostructure and plate-WO_3_ microstructures (D-WO_3_ and P-WO_3_, respectively) exhibited higher power conversion efficiency than those with a gel electrolyte. In this study, D-WO_3_ and P-WO_3_ were prepared using a hydrothermal process and their structural and morphological features were investigated for application in solar energy conversion devices. The P-WO_3_ solid-state electrolyte significantly enhanced the cell performance owing to its charge transportation and light-scattering characteristics. The P-WO_3_ solid-state electrolyte showed a power conversion efficiency of 6.3%, which is higher than those of the gel (4.2%) and D-WO_3_ solid-state (5.5%) electrolytes. The electro-chemical impedance spectroscopy (EIS), intensity-modulated voltage spectroscopy (IMVS), diffuse reflectance, and incident photon-to-current conversion efficiency (IPCE) analysis results showed that the P-WO_3_ solid-state electrolyte showed improved charge transportation and light scattering, and hence enhanced the cell performance.

## 1. Introduction

As an alternative to fossil fuel-based devices, dye-sensitized solar cells (DSSCs) are considered as one of the promising next-generation energy devices. DSSCs can be prepared easily at a low cost [1]. Among the electrolytes used in DSSCs, liquid-state electrolytes have been studied extensively due to their high efficiency (~12%) [2,3,4,5,6,7,8,9,10]. However, liquid-state electrolytes exhibit instability because of leakage and evaporation, and hence find limited DSSC applications.

Owing to their thermal and long-term stability, solvent independence, and environmental safety, solid-state electrolytes have gained significant attention [11,12,13,14,15,16]. However, solid-state electrolytes with highly crystalline polymers show lower mobility and ionic conductivity than liquid-state electrolytes. To reduce the polymer crystallization, additional inorganic nanofillers can be introduced [17]. In addition, this improves the free volume, thereby improving the ion conductivity and mobility of the solid electrolyte [18].

Introduction of the light-scattering effect is a simple method to enhance the light-harvesting capacity of solid-state DSSCs (ssDSSCs). Various studies have been carried out on introducing light scattering or photonic crystal layers within the photoanode [19,20]. On the other hand, at the photoanode, the nanomaterial-based light-scattering layer can undergo delayed charge transport due to the large number of grain boundaries, which limits the photovoltaic efficiency of ssDSSC. Hence, the use of nano or microstructured electrolytes is an efficient way to enhance the light-harvesting properties and to improve the short-circuit current density of ssDSSCs without inducing charge recombination.

In this study, we prepared a solid-state electrolyte consisting of disk-WO_3_ (D-WO_3_) nanostructures and plate-WO_3_ (P-WO_3_) microstructures for ssDSSCs. The D-WO_3_ nanostructures and P-WO_3_ microstructures were fabricated through the hydrothermal process. Manufactured D-WO3 and P-WO3 solid-state electrolytes were thoroughly characterized for material characteristics through scanning electron microscopy (SEM) and wide-angle X-ray scattering (WAXS), and for photovoltaic efficiencies by electrochemical impedance spectroscopy (EIS), incident photon-current conversion efficiency (IPCE) analysis, diffuse reflectance analysis, and intensity-modulated voltage spectroscopy (IMVS). As a control, a gel electrolyte was prepared without D-WO_3_ nanostructures or P-WO_3_ microstructures.

## 2. Experiment

### 2.1. Materials

Titanium isopropoxide, 4-tert-butylpyridine, poly (ethylene glycol) (PEG, *M_n_* = 10000 g/mol), lithium iodide (LiI), chloroplatinic acid, sodium tungsten dihydrate (Na_2_WO_4_·2H_2_O), 1-methyl-3-propylimidazolium iodide (MPII), I_2_, ammonium oxalate ((NH_4_)_2_C_2_O_4_), and titanium diisopropoxide bis (acetylacetonate) 75 wt% (Ti(acac)_2_OiPr_2_) were purchased from Sigma Aldrich (St Louis, MO, USA). Commercial nano-TiO_2_ paste (Ti-Nanoxide, D20) and ruthenium (Ru) sensitizer (N719) were purchased from Solaronix (Aubonne, Switzerland). Acetonitrile, ethyl alcohol, chloroform, and 2-propanol were purchased from J.T. Baker. Fluorine doped tin oxide (F-SnO_2_)-conducting substrates (FTO glass, TEC7) were purchased from Pilkington (Sandouville, France). All the chemicals and solvents were as received.

### 2.2. Preparation of Disk-WO_3_ (D-WO_3_) Nanostructures

The D-WO_3_ nanostructures were synthesized via a facile one-step hydrothermal process. In brief, 0.79 g of Na_2_WO_4_·2H_2_O was dissolved in 45 mL of deionized water (>18 MΩm) at 25 °C. Then, 1 mL of 10 M HCl was added to the as-prepared solution under constant stirring followed by the introduction of 0.2 g of ammonium oxalate ((NH_4_)_2_C_2_O_4_). After 2 min of stirring, 30 mL of deionized water (>18 MΩm) was added to the reaction mixture with stirring for 30 min. The as-prepared D-WO_3_ precursor solution was transferred and heated for 3 h at 140 °C in a 100 mL Teflon-lined stainless-steel autoclave reactor. The as obtained precipitate was then thermally treated at 400 °C for 2 h in air condition to obtain the D-WO_3_ nanostructures.

### 2.3. Preparation of Plate-WO_3_ (P-WO_3_) Microstructures

The P-WO_3_ microstructures were prepared using a facile one-step hydrothermal process. In brief, 0.231 g of Na_2_WO_4_·2H_2_O was dissolved in 30 mL of deionized water (>18 MΩm) under stirring at 25 °C followed by the introduction of 10 mL of 3 M HCl, resulting in a precipitate. After stirring for 30 min, the resulting solution was transferred and heated for 3 h at 140 °C in a 100 mL Teflon-lined stainless-steel autoclave reactor. The precipitate was filtered, washed with distilled water and ethanol several times to reduce the ions possibly remaining in the products, and was dried in air. The as obtained precipitate was then thermally treated at 400 °C for 2 h in air condition to obtain the P-WO_3_ microstructures.

### 2.4. Preparation of the D-WO_3_ and P-WO_3_ Solid-State Electrolytes

The D-WO_3_ and P-WO_3_ solid-state electrolytes were prepared using the solution-casting method. First, 1g of PEG was dissolved in 10 mL of acetonitrile. The concentrations of D-WO_3_ and P-WO_3_ were fixed at 1 wt% relative to PEG. The molar ratio of the ether group of PEG to two iodine salts (LiI, MPII) was fixed at 20, and the iodine (I_2_) content was fixed at 10 wt% with respect to the iodine salt (See Table S3). The solvent (acetonitrile) was completely evaporated by vacuum drying. As a control sample, a gel electrolyte was prepared using the same procedure without the D-WO_3_ nanostructures and P-WO_3_ microstructures.

### 2.5. Fabrication of Dye-Sensitized Solar Cells (DSSCs)

DSSCs with an active area of 0.4 cm^2^ were prepared. A transparent FTO substrate was employed to prepare both the two electrodes (photoanodes and counter electrodes). For the photoanode, a Ti (IV) bis(ethyl acetoacetate) diisopropoxide solution was first spin-coated onto the FTO substrate and was then annealed stepwise to 450 °C and maintained at this temperature for 30 min. Then, a commercial nano-TiO_2_ paste (Ti-Nanoxide, D20) was cast onto the FTO substrate using the doctor blade method followed by annealing at 450 °C for 30 min. The nanocrystalline TiO_2_ photoanode (10 μm thickness) was sensitized overnight with 13 mg of a ruthenium dye (535-bisTBA, N719) dissolved in distilled ethanol (50 mL). Counter electrodes were prepared by spin coating a solution of 1 wt% H_2_PtCl_6_ in isopropanol onto the FTO substrate followed by annealed at 450 °C for 30 min. A dilute gel or solid-state electrolyte solution was cast onto a dye-adsorbed TiO_2_ photoanode and was then evaporated for easy penetration through the nanopores of the TiO_2_ photoanode. Then, a concentrated gel or solid-state electrolyte solution was cast onto the photoanode to minimize the time required for solvent evaporation and to prevent the formation of cavities between the photoanode and the counter electrode during the solvent evaporation. Then, both the photoanode and the counter electrodes were superposed together and were pressed between two glass plates in order to achieve slow solvent evaporation and to obtain a thin electrolyte layer. The cells were placed in a vacuum oven for the complete evaporation of the solvent for 24 h and were then sealed with an epoxy resin.

### 2.6. Characterization

The surface morphologies of the D-WO_3_ nanostructures and P-WO_3_ microstructures were observed using scanning electron microscopy (SEM) (Hitachi SU 8010, Hitachi High-Technologies Corporation, Abingdon, UK). Wide angle X-ray diffraction (WAXs) was carried out using a Rigaku 3-kW rotating-anode X-ray generator with Cu Kα radiation (λ = 1.5406 Å) operating at 30 kV and 100 mA. The two theta range was 15–65° and a scanning speed of 10°/min was used. Electrochemical impedance spectroscopy (EIS) measurements were carried out at the open-circuit voltage (*V_oc_*) over the frequency range of 10^−2^–10^6^ Hz with an amplitude of 10 mV. The incident photo to electron conversion efficiency (IPCE) spectra of the D-WO_3_ nanostructures and P-WO_3_ microstructures were obtained using a CE system (IVIUM Technologies, Eindhoven, Netherlands). The diffused reflectance spectra of the D-WO_3_ nanostructures and P-WO_3_ microstructures were recorded over the wavelength range of 400–800 nm using a ultraviolet (UV)-visible spectrophotometer (Hewlett-Packard, Hayward, CA, USA). Intensity-modulated voltage spectroscopy (IMVS) measurements were carried out on an electrochemical workstation equipped with a frequency response analyzer under a modulated red-light emitting diode (635 nm) driven by a source supply, which could provide both the direct current/alternating current (DC/AC) components of the illumination. Frequency was varied from 10^−2^ to 10^4^ Hz. Current density-voltage (*J-V*) measurements were carried out on a potentiostat (IVIUM Technologies, Eindhoven, Netherlands). The photovoltaic parameters of the nano and microstructures were determined using the following equations:

*FF* = *V*_max_*J*_max_/*V*_oc_*J*_sc_ (1)

*η* = *V*_max_*J*_max_/*Pin* 100 = *V*_oc_*J*_sc_*FF*/*P*_in_ 100 (2)

where *η* is the solar power conversion efficiency (%), *J*_sc_ is the short circuit current density, *V*_oc_ is the open circuit voltage, *P*_in_ is the incident solar light power, and *FF* is the fill factor. *J*_max_ and *V*_max_ are the current density and voltage in the *J–V* curves of the structures at the maximum solar power output, respectively.

## 3. Results and Discussion

**Scheme 1:**
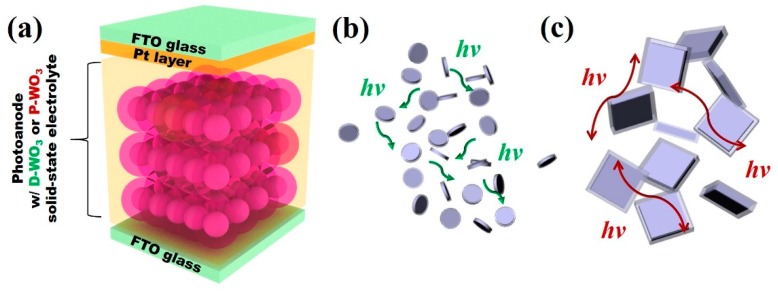
(**a**) Schematic of the dye-sensitized solar cells (DSSCs) fabricated in this study and the possible light scattering mechanisms of the (**b**) disk-WO_3_ (D-WO_3_) and (**c**) plate-WO_3_ (P-WO_3_) solid-state electrolytes.

To improve the charge transportation and light scattering properties of the solid-state electrolyte, we employed P-WO_3_ microstructures to create charge pathways and a light-scattering center, as shown in Scheme 1. The ssDSSC performance was improved by the P-WO_3_ solid-state electrolyte, which facilitated the movement of the redox I^−^/I_3_^−^ couple and enhanced the light-scattering characteristics of the ssDSSC. In order to survey the effect of the P-WO_3_ solid-state electrolyte on the DSSC performance, gel and D-WO_3_ solid-state electrolytes were also prepared.

**Figure 1:**
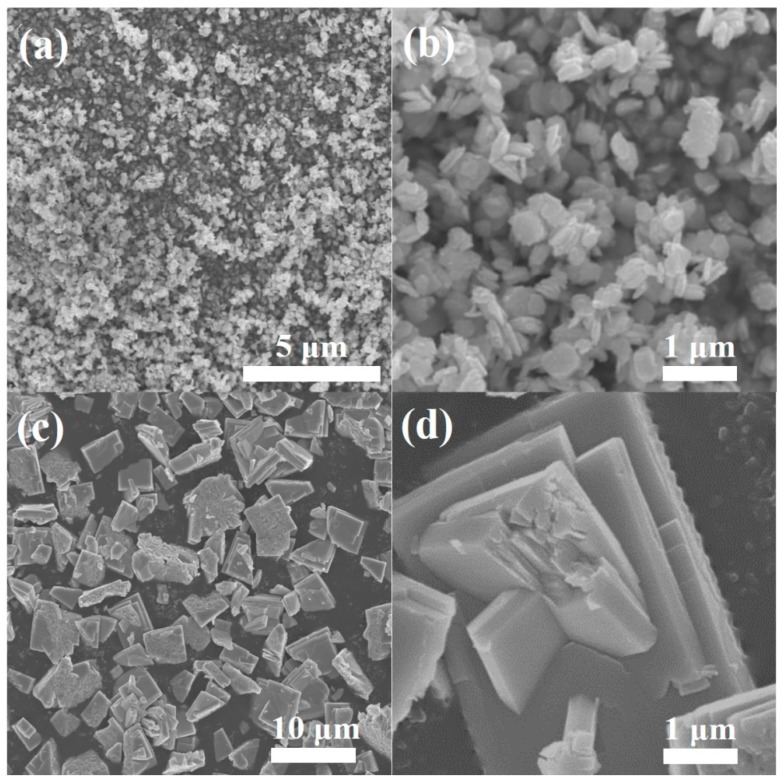
Scanning electron microscope (SEM) image of (**a**) D-WO_3,_ (**b**) magnified D-WO_3_, (**c**) P-WO_3_, and (**d**) magnified P-WO_3_.

Figure 1 exhibites the SEM image of the D-WO_3_ nanostructures and P-WO_3_ microstructures. The D-WO_3_ nanostructures showed an average diameter of 300 nm. The P-WO_3_ microstructures on the other hand, were approximately 5 μm in length. Gel electrolytes without nanofillers are transparent and show low light-scattering features. However, the P-WO_3_ solid-state electrolyte showed rough layers owing to its irregular morphology. These layers showed light-scattering properties. The diffuse reflectance plots of DSSCs fabricated with gel electrolyte, D-WO_3_ solid-state electrolytes, and P-WO_3_ solid-state electrolytes were also measured by spectrophotometer (Figure S1). Hence, the P-WO_3_ solid-state electrolyte showed better light harvesting than the gel and D-WO_3_ solid-state electrolytes.

**Figure 2:**
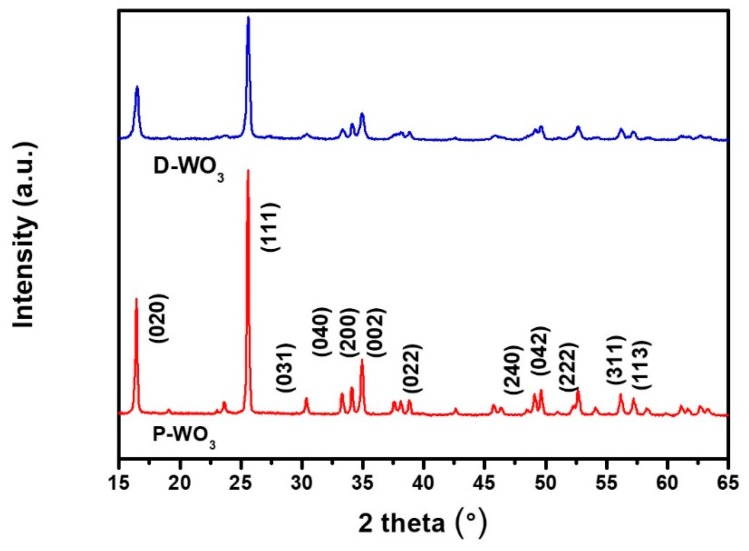
Wide-angle X-ray scattering (WAXS) results of D-WO_3_ and P-WO_3_.

The WAXS results of the D-WO_3_ nanostructures and P-WO_3_ microstructures prepared using the hydrothermal process are shown in Figure 2. To acquire more quantitative information, the average crystalline WO_3_ size was calculated using the following Scherrer equation:

*D* = *kλ/β cosθ* (3)

where *D* is the average crystalline WO_3_ size, *k* is the shape factor of the nanostructure, *λ* is the X-ray wavelength, *β* is the full width of the Bragg’s diffraction peak at half its maximum intensity, and *θ* is half of the Bragg’s diffraction angle of the center of the peak [13]. Both the D-WO_3_ nanostructures and P-WO_3_ microstructures exhibited WAXS peaks at 2*θ* = 16.4, 25.6, 30.4, 33.3, 34.1, 35.0, 38.8, 48.5, 49.1, 49.6, 52.6, 56.1, and 57.2 corresponding to the (002), (111), (031), (040), (200), (002), (022), (240), (042), (222), (311), and (113) planes, respectively. These peaks could be indexed to the typical pattern of orthorhombic WO_3_ (JCPDS No. 43-0679) [21]. These results confirm the successful preparation of highly crystalline orthorhombic D-WO_3_ nanostructures and P-WO_3_ microstructures from the WO_3_ precursor using the hydrothermal process. All the WAXS peaks of the D-WO_3_ nanostructures synthesized via the hydrothermal process were relatively wide, indicating that these nanostructures showed smaller crystallite size than the P-WO_3_ microstructures. The average WO_3_ crystallite size of the D-WO_3_ nanostructures was estimated to be approximately 300 nm (using Equation (3)). This result is consistent with the nanostructure size obtained from the previous SEM image.

**Figure 3:**
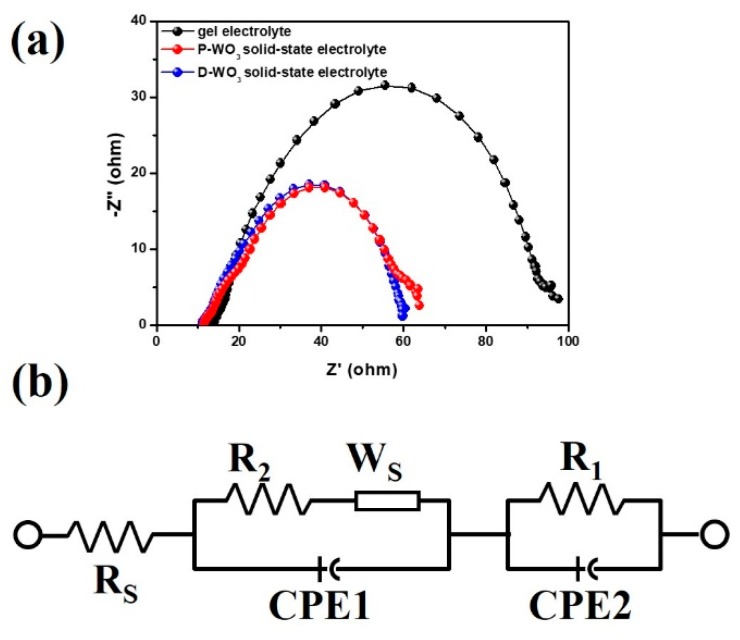
(**a**) Electro-chemical impedance spectroscopy (EIS) Nyquist plots of the DSSCs fabricated with the gel, D-WO_3_ solid-state, and P-WO_3_ solid-state electrolytes obtained under one sun illumination (AM 1.5, 100 mW cm^−2^) and (**b**) equivalent circuit of DSSCs.

EIS analysis was carried out at frequencies ranging from 10^−2^ Hz to 10^−1^ MHz with an AC amplitude of 0.02 V under 1 sun illumination. Figure 3a,b show the Nyquist plots and the equivalent circuit for the EIS spectra of the ssDSSCs employing the gel, D-WO_3_ solid-state, and P-WO_3_ solid-state electrolytes, respectively. The electro-kinetic characteristics, i.e., the interfacial resistance characteristics (*Rs*, *R_1_*, *R_2_*, and *Ws*) of the ssDSSCs are summarized in Table 1. *Rs* is the series interfacial resistance including the sheet resistance of the FTO substrate, R_1_ is the charge transfer interfacial resistance of the counterelectrode, *R_2_* is the charge transfer interfacial resistance of the photoanode/sensitizer/electrolyte interface, and *Ws* is the ionic diffusion within the electrolyte. CPE1 and CPE2 are the constant phase components of capacitance corresponding to R_1_ and R_2_, respectively [22,23]. The total interfacial resistances of the ssDSSCs with the gel, D-WO_3_ solid-state, and P-WO_3_ solid-state electrolytes were 97.6, 60, and 63.9 Ω, respectively, as calculated using the EIS data fitted with the equivalent circuit. Because we used identical FTO substrates, photoanodes, and counter electrodes, the difference in the *Rs* and *R_1_* values was insignificant. Therefore, the difference in the total interfacial resistance, which was related with the *R_2_* value at the TiO_2_ photoanode/sensitizer/electrolyte interface, of the three devices can be mainly assiciated to the solid-state electrolytes based on D-WO_3_ and P-WO_3_. The charge transfer interfacial resistances of the D-WO_3_ (*R_2_* = 42.9 Ω) and P-WO_3_ (*R_2_* = 43.4 Ω) solid-state electrolytes were lower than that of the gel electrolyte (*R_2_* = 76.9 Ω). This can be attributed to the enhancement in the continuous free volumes related to ion-mobility resulting from the interaction between the WO_3_ nano or microstructures and the highly crystalline polymer chain. In addition, the ionic diffusions within the P-WO_3_ solid-state electrolyte (*Ws*) were slightly larger than those of the other systems. This result indicates that the viscosity of the P-WO_3_ solid-state electrolyte was higher than those of the other electrolytes. Similar results have been reported previously [24]. To further understand the dynamics of charge transport, the recombination kinetics of the three types of cells were characterized by using dark EIS and IMVS (Figures S2 and S3).

**Figure 4:**
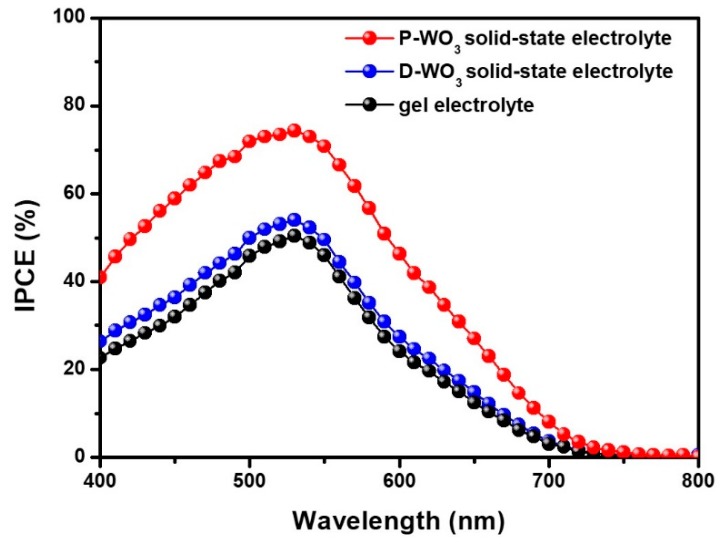
IPCE plots of the DSSCs fabricated with the gel, D-WO_3_ solid-state, and P-WO_3_ solid-state electrolytes.

The IPCE analysis of the ssDSSCs provided quantitative information on their light-harvesting properties. Figure 4 exhibits the IPCE analysis of the ssDSSCs based on the gel, D-WO_3_ solid-state, and P-WO_3_ solid-state electrolytes. The P-WO_3_ solid-state electrolyte-based DSSC exhibited a maximum IPCE of 72% at a wavelength of about 540 nm while the gel electrolyte- and D-WO_3_ solid-state electrolyte-based ssDSSCs showed the IPCEs of 45% and 52%, respectively. This indicates that the P-WO_3_ solid-state electrolyte acted as an effective light-scattering center to enhance the scattering of the unabsorbed solar light back into the DSSC. Therefore, the P-WO_3_ solid-state electrolyte improved the light-harvesting capability of the DSSC through efficient utilization of incident light.

**Figure 5:**
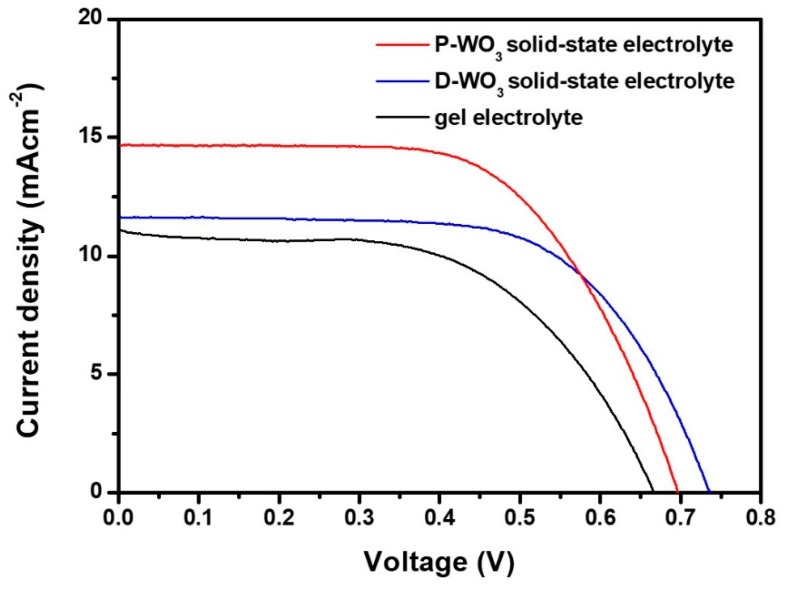
Current density-voltage (*J-V*) curves of the DSSCs fabricated with the gel, D-WO_3_ solid-state, and P-WO_3_ solid-state electrolytes obtained under one sun illumination (AM 1.5, 100 mW cm^−2^).

Solid-state electrolytes with D-WO_3_ nanostructures and P-WO_3_ microstructures were prepared for fabricating DSSCs. The photovoltaic properties of the D-WO_3_ and P-WO_3_ solid-state electrolyte-based DSSCs are shown in Figure 5. Also, the performance of the liquid electrolyte was evaluated in DSSCs under on sun (AM 1.5) illumination (Figure 4, Table S2). The photovoltaic characteristics including the open-circuit voltage (*Voc*), short-circuit current density (*Jsc*), fill factor (*FF*), and solar power conversion efficiency (*η*) of the DSSCs are listed in Table 1. The measurements were carried out under one sun illumination. As a control, a gel electrolyte was prepared using the same procedure without D-WO_3_ nanostructures or P-WO_3_ microstructures. The solid-state nanofiller ratio (1 wt%) was minimized to survey the effect of the morphology of the D-WO_3_ nanostructures and P-WO_3_ microstructures on the DSSC performance. The *Jsc* value of a device can be estimated by integrating its light harvesting, electron injection, and electron collection efficiencies using the following equation:

*Jsc* = *qη_lh_η_inj_·η_col_I_0_* (4)

where *q* is the elementary charge, *η_lh_* is the light harvesting efficiency, *η_inj_* is the electron injection efficiency, *η_col_* is the electron collection efficiency, and *I_0_* is the solar light flux [25,26]. The solar power conversion efficiencies of the ssDSSCs prepared with the D-WO_3_ nanostructure- and P-WO_3_ microstructure-based solid-state electrolytes were found to be 5.5 and 6.3%, at 100 mW/cm^2^, which is one of the highest values for ssDSSCs (Table S1). These values are approximately 1.3- and 1.5-fold higher than that of the gel electrolyte-based DSSC, respectively. This result demonstrates the importance of incorporating inorganic WO_3_ nanofillers in solid-state electrolytes. This enhancement in the DSSC performance with the incorporation the WO_3_ nanofillers can be attributed to their high *Jsc* and *FF* values. According to Equation (4), light-harvesting characteristics were responsible for the high *Jsc* value of the ssDSSC based on the P-WO_3_ solid-state electrolyte. Owing to the light-harvesting characteristics of P-WO_3_, the light utilization efficiency of the ssDSSC improved along with an improve in the *Jsc* value. These results are consistent with the IPCE results shown in Figure 4. In addition, the D-WO_3_ nanostructures and P-WO_3_ microstructures acted as nanofillers and decreased the crystallinity of the polymer electrolyte matrix, thus enhancing the continuous free volumes, charge-carrier mobility, and *FF* values of the ssDSSCs.

## 4. Conclusions

The solar power conversion efficiency of the P-WO_3_ solid-state electrolyte-based ssDSSC was higher than those of the gel electrolyte- and D-WO_3_ solid-state electrolyte-based DSSCs. The ssDSSC fabricated with the P-WO_3_ solid-state electrolyte showed a solar power conversion efficiency of 6.3%, which is higher than those of the DSSCs with the gel (4.2%) and D-WO_3_ solid-state (5.5%) electrolytes. P-WO_3_ solid-state electrolytes offer enhanced charge transportation and light-scattering characteristics, can confine the incident light within the electrolyte/electrode, and limit the interfacial resistance, thereby increasing the cell performance. The results exhibited that the introduction of nano or microstructures within the non-electrode elements of DSSCs such as electrolytes can enhance the light-harvesting characteristic of the cells avoiding charge recombination, and hence increase their solar-energy conversion performance.

## Figures and Tables

**Table 1:** Photovoltaic and electrochemical properties of the DSSCs and ssDSSCs fabricated with the gel, D-WO_3_ solid-state, and P-WO_3_ solid-state electrolytes under 1 sun illumination (AM 1.5, 100 mW cm^−2^) ^a^.

Electrolyte	*V_oc_* (V)	*J_sc_*^b^ (mA/cm^2^)	*J_sc_*^c^ (mA/cm^2^)	*FF*	*η* (%)	*R_S_*	*R_1_*	*R_2_*	*W_S_*
Gel	0.67 ± 0.2	11.1 ± 0.3	11.0	0.56 ± 0.02	4.2 ± 0.3	13.7	2.1	76.9	4.9
D-WO_3_ solid-state	0.74 ± 0.1	11.6 ± 0.3	11.8	0.64 ± 0.02	5.5 ± 0.2	11	2.5	42.9	3.6
P-WO_3_ solid-state	0.71 ± 0.1	14.6 ± 0.2	14.3	0.61 ± 0.02	6.3 ± 0.2	11.5	2.2	43.4	6.8

^a^ A typical dye-sensitized solar cell had an active area of 0.40/cm^2^ and was masked using an aperture with similar area during the solar cell performance measurements. The thickness of the TiO_2_ photoanode was about 7 µm. ^b^ Determined from the J-V curves. ^c^ Determined from the incident photon-to-current conversion efficiency (IPCE) curves.

## Supplementary Materials

The following are available online at https://www.mdpi.com/2079-4991/9/12/1797/s1, Figure S1: Diffuse reflectance plots of DSSCs fabricated with gel electrolyte, D-WO_3_ solid-state electrolytes, and P-WO_3_ solid-state electrolytes, Figure S2: EIS curves of DSSCs fabricated with gel electrolyte, D-WO_3_ solid-state electrolytes, and P-WO_3_ solid-state electrolytes measured at -0.65 V bias voltage in dark condition (100 kHz~10 mHz), Figure S3: IMVS of DSSCs fabricated with gel electrolyte, D-WO_3_ solid-state electrolytes, and P-WO_3_ solid-state electrolytes, Figure S4: J-V curves DSSCs fabricated with liquid electrolyte and P-WO_3_ solid-state electrolytes that were obtained under one sun illumination (AM 1.5, 100 mW cm^−2^), Table S1: Comparison of photovoltaic parameters of DSSCs fabricated with polymer gel or solid-state electrolytes reported in the literature, Table S2: Photovoltaic parameters of DSSCs fabricated with liquid electrolyte and P-WO_3_ solid-state electrolytes that were obtained under one sun illumination (AM 1.5, 100 mW cm^−2^), Table S3: DSSCs electrolyte formulations.
