# Supplementary material for: Solid-State Solar Energy Conversion from WO3 Nano and Microstructures with Charge Transportation and Light-Scattering Characteristics
Source: Nanomaterials (Basel). 2019 Dec 17;9(12):1797. doi: 10.3390/nano9121797 (PMC6956145; doi:10.3390/nano9121797)
Supplement: Supplementary file 1 [file nanomaterials-09-01797-s001.pdf]

Solid-State Solar Energy Conversion from WO_3 Nano and Microstructures with Charge Transportation and Light-Scattering Characteristics

Juyoung Moon [1,†], Woojun Shin [2,†], Jung Tae Park [1,*] and Hongje Jang [2,*]

[1] Department of Chemical Engineering, Konkuk University, 120 Neungdong-ro, Gwangjin-gu, Seoul 05029, Korea

[2] Department of Chemistry, Kwangwoon University, 20 Gwangwoon-ro, Nowon-gu, Seoul 01897, Korea

* Correspondence: jtpark25@konkuk.ac.kr (J.T.P.); hjang@kw.ac.kr (H.J.); Tel: +82-2-450-3538 (J.T.P.); +82-2-940-8320 (H.J.)

† These authors contributed equally to this work.

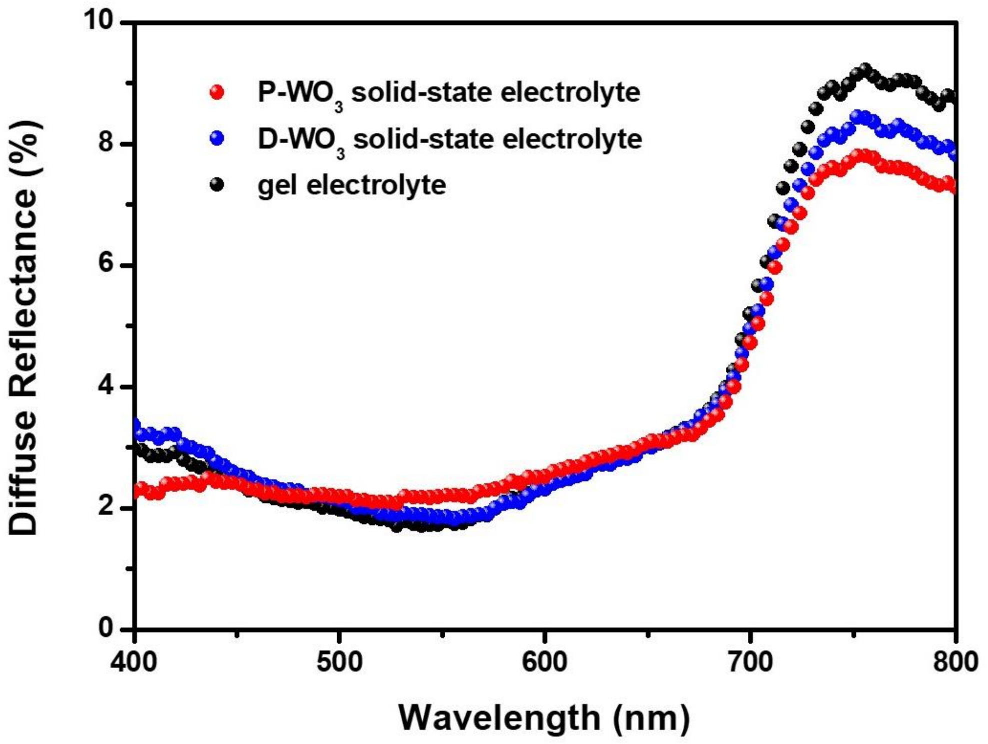

Figure S1. Diffuse reflectance plots of DSSCs fabricated with gel electrolyte, D-WO_3 solid-state electrolytes, and P-WO_3 solid-state electrolytes.

The amount of diffusely scattered light in D-WO_3 nanostructures and P-WO3 microstructures based electrolytes as a result of a beam of irradiation on the ssDSSCs, can be quantified by diffuse reflectance spectroscopy. Compared to the gel electrolyte based DSSCs, the ssDSSCs composed of P-WO_3 solid-state electrolytes had a little higher diffuse reflection values in the visible light regions. This result indicating that the incident light was well scattered within the P-WO_3 solid-state electrolytes than gel electrolytes. Also, it should be noted that the presence of not only the electrolyte but also the TiO_2 layer, sensitizer, FTO substrate in devices, leading to a decrease in the difference of diffuse reflectance values, as a similar result was reported previously [1–3]. Therefore, we believe the diffuse reflectance analysis of different electrolyte systems including the TiO_2 layer, sensitizer, and FTO substrate strongly supports our results.

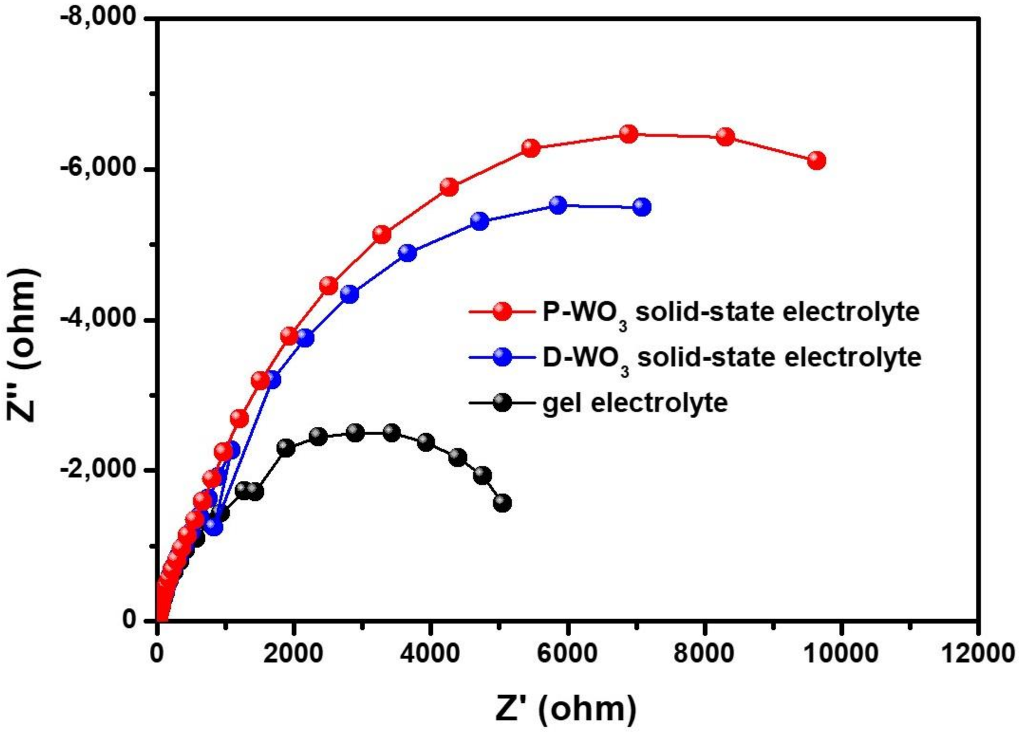

Figure S2. EIS curves of DSSCs fabricated with gel electrolyte, D-WO_3 solid-state electrolytes, and P-WO_3 solid-state electrolytes measured at -0.65 V bias voltage in dark condition (100 kHz ~ 10 mHz).

The suppression of the electron recombination process by the P-WO_3 solid-state electrolyte was confirmed by EIS curves of ssDSSCs measured under dark conditions. As a result, there was an improvement in the open-circuit voltage (*Voc*) for P-WO_3 solid-state electrolyte, which results from the slower recapture of conduction band electrons by I_3^-, reduced interfacial charge recombination loss and enhanced electron transport.

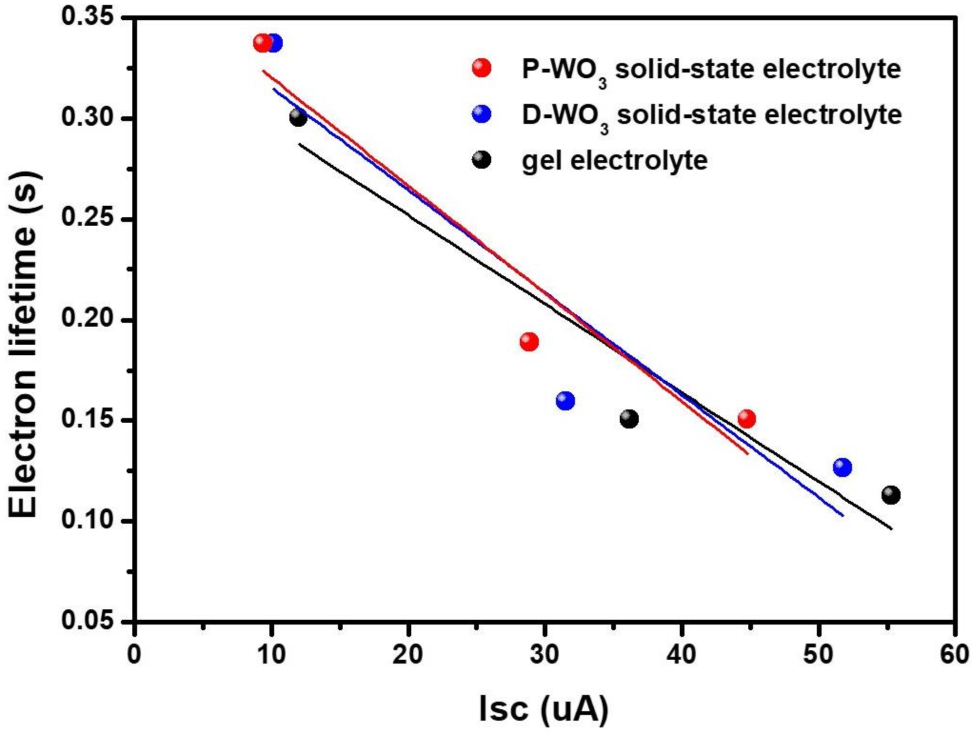

Figure S3. IMVS of DSSCs fabricated with gel electrolyte, D-WO_3 solid-state electrolytes, and P-WO_3 solid-state electrolytes.

Figure S3 shows that the electron lifetime values of the P-WO_3 solid-state electrolytes based ssDSSCs are greater than those of the gel electrolytes. This result indicates enhanced electron transport rate and reduced recombination or back reaction in the P-WO_3 solid-state electrolytes based ssDSSCs than the gel electrolytes system.

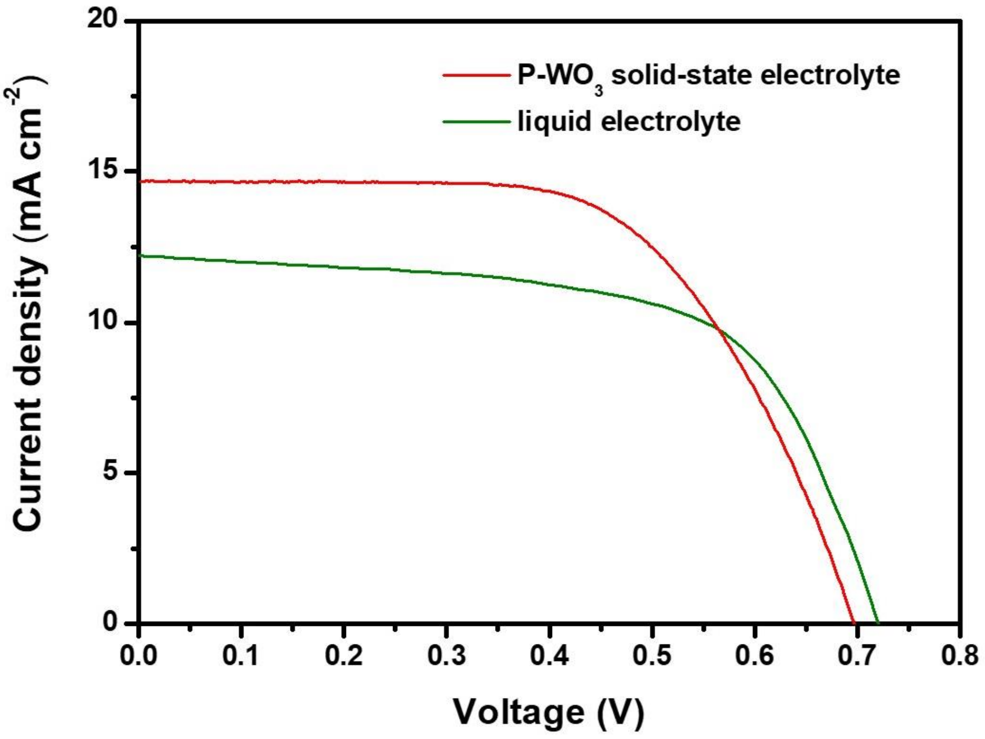

Figure S4. J-V curves DSSCs fabricated with liquid electrolyte and P-WO_3 solid-state electrolytes that were obtained under one sun illumination (AM 1.5, 100mW cm^{-2}).[a]

[a] Liquid electrolyte consisting of 1-butyl-3-methylimidazolium iodide, I_2, guanidinium thiocyanate, and 4-tert-butylpyridine in a mixture of acetonitrile and valeronitrile.

Our results show that ssDSSCs generated with a P-WO_3 solid-state electrolyte has a power conversion efficiency of 6.8%, which is higher than that of DSSCs using a common liquid electrolyte (5.5%).

Table S1. Comparison of photovoltaic parameters of DSSCs fabricated with polymer gel or solid-state electrolytes reported in the literature.

Electrolyte	V_{oc} (V)	J_{sc} (mA/cm^2)	*FF*	η (%)	Reference
P-WO_3	0.71	14.6	0.61	6.3	This work
PEO/PEGDME	0.79	12.6	0.77	7.7	[4]
P-CNT-5	0.65	22.0	0.62	8.9	[5]
MOG	0.70	12.9	0.72	6.5	[6]
Unitary	0.60	15.5	0.65	6.1	[7]
Zeolite-XF12	0.74	13.7	0.60	6.0	[8]

The device efficiency of the DSSCs using the P-WO_3 solid-state electrolyte reached 6.3% at 100 mW cm^{-2}, which is again higher than that (4.2%) of the gel electrolyte based system and represents one of the highest values reported for gel or solid-state DSSCs to date [4–8].

Table S2. Photovoltaic parameters of DSSCs fabricated with liquid electrolyte and P-WO_3 solid-state electrolytes that were obtained under one sun illumination (AM 1.5, 100mW cm^{-2}).[a]

Electrolyte	V_{oc} (V)	J_{sc} (mA/cm^2)	*FF*	η (%)
liquid	0.71	12.2	0.64	5.5
P-WO_3 solid-state	0.71	14.6	0.61	6.3

[a] A typical dye-sensitized solar cells had an active area of ca. 0.40 cm^{-2} and was masked using an aperture of the identical area during the *J-V* measurements. And, thickness of the photoanode was approximately 10 μm.

Table S3. DSSCs electrolyte formulations.

Electrolyte	WO_3	PEG	LiI	MPII	I_2	Acetonitrile
gel	-	1 g	0.15 g	0.15 g	0.03 g	10 mL
D-WO_3 solid-state	0.01 g	1 mL	0.15g	0.15 g	0.03 g	10 mL
P-WO_3 solid-state	0.01 g	1 mL	0.15g	0.15 g	0.03 g	10 mL

References

1. Bharwal, A. K.; Manceriu, L.; Alloin, F.; Iojoiu, C.; Dewalque, J.; Toupance, T.; Henrist, C. Bimodal titanium oxide photoelectrodes with tuned porosity for improved light harvesting and polysiloxane-based polymer electrolyte infiltration, *Sol. Energy*, **2019**, 178, 98–107.
2. Xia, W.; Mei, C.; Zeng, X.; Chang, S.; Wu, G.; Shen, X. Mesoporous multi-shelled ZnO microspheres for the scattering layer of dye sensitized solar cell with a high efficiency, *Appl. Phys. Lett.* **2016**, 108, 113902.
3. Zijian, C.; Kaiyue, Z.; Guangyu, X.; Yaqing, F.; Shuxian, M. Multi-functional 3D N-doped TiO_2 microspheres used as scattering layers for dye-sensitized solar cells, *Front. Chem. Sci. Eng.* **2017**, 11, 395–404.
4. Li, C.; Xin, C.; Xu, L.; Zhong, Y.; Wu, W. Components control for high-voltage quasi-solid state dye-sensitized solar cells based on two-phase polymer gel electrolyte, *Sol. Energy*, **2019**, 181, 130–136.
5. Sakali, S. M.; Khanmirzaei, M. H.; Lu, S. C.; Ramesh, S.; Ramesh, K. Investigation on gel polymer electrolyte-based dye-sensitized solar cells using carbon nanotube, *Ionics*, **2019**, 25, 319–325.
6. Zhang, W.; Wang, Z.; Tao, L.; Duan, K.; Wang, H.; Zhang, J.; Pan, X.; Huo, Z. A promising heat-induced supramolecular metallogel electrolyte for quasi-solid-state dye-sensitized solar cells, *J. Solid State Electrochem.*, **2019**, 23, 1563–1570.
7. Tao, L.; Zhang, W.; Wang, Z.; Wang, H.; Zhang, J.; Huo, Z.; Dai, S.; Hayat, T.; Alharbi, N.S. Highly improved photocurrent and stability of dye-sensitized solar cell through quasi-solid-state electrolyte formed by two low molecular mass organogelators, *Org. Electron.*, **2019**, *65*, 179–184.
8. Lim, J. M.; Park, J.; Park, J.T.; Bae, S. Preparation of quasi-solid-state electrolytes using a coal fly ash derived zeolite-X and -A for dye-sensitized solar cells, *J. Ind. Eng. Chem.*, **2019**, *71*, 378–386.
